# Insular dysfunction within the salience network is associated with severity of symptoms and aberrant inter-network connectivity in major depressive disorder

**DOI:** 10.3389/fnhum.2013.00930

**Published:** 2014-01-21

**Authors:** Andrei Manoliu, Chun Meng, Felix Brandl, Anselm Doll, Masoud Tahmasian, Martin Scherr, Dirk Schwerthöffer, Claus Zimmer, Hans Förstl, Josef Bäuml, Valentin Riedl, Afra M. Wohlschläger, Christian Sorg

**Affiliations:** ^1^Department of Psychiatry, Klinikum Rechts der Isar, Technische Universität MünchenMunich, Germany; ^2^Department of Neuroradiology, Klinikum Rechts der Isar, Technische Universität MünchenMunich, Germany; ^3^TUM-Neuroimaging Center, Technische Universität MünchenMunich, Germany; ^4^Department of Radiology, University Hospital ZürichZürich, Switzerland; ^5^Munich Center for Neurosciences Brain & Mind, Ludwig-Maximilians-Universität MünchenMunich, Germany; ^6^Department of Neurology, Christian Doppler Klinik, Paracelsus Medical University SalzburgSalzburg, Austria; ^7^Department of Nuclear Medicine, Klinikum Rechts der Isar, Technische Universität MünchenMunich, Germany

**Keywords:** intrinsic functional connectivity, intrinsic networks, central executive network, default mode network, salience network, triple network hypothesis, anterior insula, major depressive disorder

## Abstract

Major depressive disorder (MDD) is characterized by altered intrinsic functional connectivity within (intra-iFC) intrinsic connectivity networks (ICNs), such as the Default Mode- (DMN), Salience- (SN) and Central Executive Network (CEN). It has been proposed that aberrant switching between DMN-mediated self-referential and CEN-mediated goal-directed cognitive processes might contribute to MDD, possibly explaining patients' difficulties to disengage the processing of self-focused, often negatively biased thoughts. Recently, it has been shown that the right anterior insula (rAI) within the SN is modulating DMN/CEN interactions. Since structural and functional alterations within the AI have been frequently reported in MDD, we hypothesized that aberrant intra-iFC in the SN's rAI is associated with both aberrant iFC between DMN and CEN (inter-iFC) and severity of symptoms in MDD. Twenty-five patients with MDD and 25 healthy controls were assessed using resting-state fMRI (rs-fMRI) and psychometric examination. High-model-order independent component analysis (ICA) of rs-fMRI data was performed to identify ICNs including DMN, SN, and CEN. Intra-iFC within and inter-iFC between distinct subsystems of the DMN, SN, and CEN were calculated, compared between groups and correlated with the severity of symptoms. Patients with MDD showed (1) decreased intra-iFC within the SN's rAI, (2) decreased inter-iFC between the DMN and CEN, and (3) increased inter-iFC between the SN and DMN. Moreover, decreased intra-iFC in the SN's rAI was associated with severity of symptoms and aberrant DMN/CEN interactions, with the latter losing significance after correction for multiple comparisons. Our results provide evidence for a relationship between aberrant intra-iFC in the salience network's rAI, aberrant DMN/CEN interactions and severity of symptoms, suggesting a link between aberrant salience mapping, abnormal coordination of DMN/CEN based cognitive processes and psychopathology in MDD.

## Introduction

Major depressive disorder (MDD) is a severe mental disorder defined by the presence of at least one major depressive episode (MDE), which is primarily characterized by depressed mood, diminished interest, loss of energy, impaired cognition, and suicidal tendency (American Psychiatric Association, 2000). MDEs have been demonstrated to be associated with both structural (Savitz and Drevets, 2009) and functional brain anomalies including aberrant functional connectivity (FC) of remote brain areas' activity (Greicius et al., 2007; Sheline et al., 2010; Northoff et al., 2011). Altered intrinsic FC (iFC, i.e., synchronous ongoing brain activity) has been found in intrinsic connectivity networks (ICNs) particularly in the Default Mode Network (DMN), Salience Network (SN), and Central Executive Network (CEN), suggesting a critical role of these neurocognitive “core” networks (Uddin et al., 2011) in mediating pathophysiological mechanisms in MDD (Menon, 2011; Hamilton et al., 2013).

The DMN comprises mainly the ventromedial prefrontal cortex, the posterior cingulate cortex, bilateral inferior parietal cortex and the middle temporal lobe and is involved in self-referential/internally oriented processes (Buckner et al., 2008). Within the DMN, aberrant deactivation during goal-directed tasks (Sheline et al., 2009) as well as increased FC during rest [(Greicius et al., 2007), see also Broyd et al. (2009) for review] have been demonstrated in patients with MDD, suggesting that DMN-mediated processes bias patients for increased self-referential thoughts even during external tasks in MDD (Hamilton et al., 2013). The CEN comprises primarily the dorsolateral prefrontal cortex (DLPFC) and posterior parietal cortex and is involved in control processes during goal-directed/externally oriented tasks (Fox and Raichle, 2007) and regulation of emotional responses, particularly mediated via the DLPFC (Phillips et al., 2008). Heterogeneous alterations in iFC during both task (Fitzgerald et al., 2008b) and rest (Fitzgerald et al., 2008a; Diener et al., 2012) have frequently been reported in MDD, supporting the assumption of aberrant cognitive regulation of emotional processing in patients with MDD (Pizzagalli et al., 2009). The SN comprises the anterior insular cortex (AI) and dorsal anterior cingulate cortex and is involved in detecting and orienting to both external and internal salient stimuli and events (Seeley et al., 2007). The AI within the SN is critically involved in maintaining and updating representations of current and predictive salience (Singer et al., 2009; Palaniyappan and Liddle, 2012). Particularly, the right AI has been suggested to critically contribute to appropriate behavioral responses to salient stimuli via switching between DMN-related self-referential and CEN-related goal directed cognitive activity (Sridharan et al., 2008). In MDD, increased activation in response to negative stimuli (Strigo et al., 2008) as well as aberrant iFC at rest [see Sliz and Hayley (2012) and Diener et al. (2012) for extensive review] have been reported in the AI, possibly indicating heightened SN response selectively to negative stimuli. Taken together, these findings suggest a reorganization of iFC within DMN, SN and CEN in MDD, potentially contributing to characteristic symptoms in MDD, such as rumination (DMN), emotional over-reactivity (SN), and emotional disinhibition (CEN) (Hamilton et al., 2013).

Recent large-scale neurocognitive models of MDD point at the prominent role of aberrant PFC-limbic interactions underlying impaired emotion processing including its regulation in MDD (Mayberg, 1997; Drevets et al., 2008; Disner et al., 2011). In terms of intrinsic networks, these models suggest aberrant interactions among DMN, CEN, and SN, which cover large parts of the prefrontal and limbic brain (Hamilton et al., 2013). Correspondingly, Hamilton and colleagues found that increased dominance of DMN activity over the activity of the task positive network (“TPN,” which corresponds in its spatial pattern widely to the CEN) during rest is associated with the severity of self-focused rumination in depressive patients, therefore giving a first hint toward aberrant inter-network interaction and its relevance for symptoms in MDD. In line with this finding, they demonstrated further that aberrant right AI activity in patients appeared to be involved in this network interaction failure: more specifically, increased AI activity was found in patients when CEN activity increased, while in controls increased AI activity was present when DMN activity increased. This result corresponds well with right AI's critical role in modulating interactions between the DMN and CEN in healthy subjects (Sridharan et al., 2008), suggesting maladaptive interaction between right AI and DMN/CEN in patients with MDD. However, direct evidence for such aberrant inter-network interaction centered on the right AI in depression is still missing. Beyond previously reported structural and functional alterations in the AI [(Strigo et al., 2008; Sprengelmeyer et al., 2011); see also (Diener et al., 2012) for review], we hypothesized that rAI's intrinsic connectivity is aberrant and associated with both aberrant iFC between DMN and CEN (inter-iFC) and severity of symptoms in major depression.

The current study aimed to test this hypothesis by addressing the following questions: Is there a relationship between aberrant intra-iFC within and aberrant inter-iFC between intrinsic brain networks (i.e., SN, DMN, CEN) in patients with MDD? Is, as recently proposed (Menon, 2011), aberrant intra-iFC in the right AI within the SN linked to aberrant inter-iFC between the DMN and CEN? And are aberrant intra-iFC and inter-iFC not only linked to selective symptoms, such as rumination, but also to the global severity of symptoms in patients with MDD?

To investigate these questions, we performed resting-state functional magnetic resonance imaging (rs-fMRI) measuring the ongoing blood-oxygenation-level-dependent (BOLD) fluctuations, structural imaging as well as psychometric assessment in 25 patients with MDD and 25 age- and sex matched healthy controls. According to a previously reported approach (Manoliu et al., 2013b), rs-fMRI data were decomposed by high-model-order independent component analysis (ICA) into spatially independent z-maps of functionally coherent brain areas and corresponding time courses of component activity (Calhoun et al., 2001). From these spatial maps (SM), we selected those representing the DMN, SN, and CEN. Main outcome measures were Pearson's correlation between network time courses, reflecting inter-network intrinsic functional connectivity (inter-iFC) and components' z-maps, reflecting the intra-network intrinsic functional connectivity (intra-iFC). We controlled our analyses for effects of age, sex and structural anomalies.

## Methods

### Participants

Twenty-five patients with recurrent MDD and 25 healthy control subjects participated in this study (see Table 1 for detailed presentation of demographical and clinical characteristics). Participant's data have also been used in a previous study investigating the topology of the brain's connectivity patters in patients with MDD (Meng et al., 2013). All participants provided informed consent in accordance with the Human Research Committee guidelines of the Klinikum rechts der Isar, Technische Universität München. Patients were recruited from the Department of Psychiatry, healthy controls by word-of-mouth advertising. Participants' examination included medical history, psychiatric interview, psychometric assessment, and blood tests for patients. Psychiatric diagnoses were based on DSM IV (American Psychiatric Association, 2000). The Structured Clinical Interview for DSM-IV (SCID-I, Spitzer et al., 1992) was used to assess the presence of psychiatric diagnoses. Severity of clinical symptoms was measured with the Hamilton Rating Scale for Depression (HAM-D, Hamilton, 1960) as well as the Beck Depression Inventory (BDI, Beck et al., 1961). The global level of social, occupational, and psychological functioning was measured with the Global Assessment of Functioning Scale (GAF, Spitzer et al., 1992). Psychiatrists Martin Scherr and Dirk Schwerthöffer performed clinical-psychometric assessment and have been professionally trained for SCID interviews with inter-rater reliability for diagnoses and scores of more than 95%.

**Table 1:** **Demographic and clinical characteristics**.

**Measure**	**MDD (***n*** = **25**)**	**HC (***n*** = **25**)**	**MDD vs. HC^a^,^b^**
	**Mean (*SD*)**	**Mean (*SD*)**	***p*-value**
Age	48.76 (14.83)	44.08 (14.78)	>0.05^a^
Sex (m/f)	12/13	11/14	>0.05^b^
Handedness (EHI)	70.30 (21.67)	66.45 (22.04)	>0.05^a^
GAF	49.80 (10.53)	99.50 (1.10)	<0.001^a^,^*^
HAM-D	22.12 (7.06)	0	
BDI	24.08 (6.31)	0	
Duration of current MDE [weeks]	16.56 (6.62)		
Duration of illness [years]	16.72 (10.20)		
Number of MDEs	5.56 (2.47)		

a Two-sample t-test.

b χ^2^-test.

* significant for p < 0.05, Bonferroni-corrected for multiple comparisons.

Abbreviations: MDD, major depressive disorder; HC, healthy controls; SD, standard deviation; EHI, Edinburgh handedness inventory; GAF, Global Assessment of Functioning Scale; HAM-D, Hamilton Depression Rating Scale; BDI, Beck Depression Inventory.

MDD was the primary diagnosis for all patients. All patients met criteria for a current MDE with an average current episode length of 16.56 weeks (*SD* = 6.62), an averaged HAM-D score of 22.12 (*SD* = 7.06) and an average BDI score of 24.08 (*SD* = 6.31). The average GAF-score was 49.80 (*SD* = 10.53). The mean duration of MDD was 16.72 years (*SD* = 10.20), the mean number of episodes 5.56 (*SD* = 2.47). Fourteen out of twenty-five patients with MDD had a psychiatric co-morbidity, including generalized anxiety disorder (*n* = 6), somatization disorder (*n* = 3), and avoidant or dependent personality disorder (*n* = 5). Patients with psychotic symptoms, schizophrenia, schizoaffective disorder, bipolar disorder, and substance abuse were excluded from this study. Additional exclusion criteria were pregnancy, neurological or severe internal systemic diseases, and general contraindications for MRI. One patient was free of any psychotropic medication during MRI assessment. Seven patients received mono-therapy [including citalopram 30 mg/d (mean dose, *n* = 3), sertraline 200 mg/d (*n* = 3), mirtazapine 30 mg/d (*n* = 1)]. Twelve patients received dual-therapy [including citalopram 37.5 mg/d and mirtazapine 30 mg/d (*n* = 5), citalopram 40 mg/d and venlafaxine 225 mg/d (*n* = 2), citalopram 30 mg/d and quetiapine 200 mg/d (*n* = 1), sertraline 200 mg/d and mirtazapine 30 mg/d (*n* = 1), venlafaxine 225 mg/d and mirtazapine 30 mg/d (*n* = 3)]. Five patients received triple therapy [including citalopram 30 mg/d, venlafaxine 187.5 mg/d and amisulprid 200 mg/d (*n* = 2), citalopram 30 mg/d, mirtazapine 30 mg/d and quetiapine 200 mg/d (*n* = 2), venlafaxine 22 mg/d, mirtazapine 30 mg/d and quetiapine 200 mg/d (*n* = 1)]. All healthy controls were free of any current or past neurological or psychiatric disorder or psychotropic medication and had no family history of affective or psychotic mental disorders in first-degree relatives.

All participants underwent 10 min of rs-fMRI with the instruction to keep their eyes closed and not to fall asleep. We verified that subjects stayed awake and had no odd feelings during the scanning session by interrogating via intercom immediately after the rs-fMRI scan. No patient dropped out during the scanning session.

## MRI data acquisition

MRI was performed on a 3 T MR scanner (Achieva, Philips, Netherland) using an 8-channel phased-array head coil. For co-registration and volumetric analysis, T1-weighted anatomical data were obtained by using a magnetization-prepared rapid acquisition gradient echo sequence (*TE* = 4 ms, *TR* = 9 ms, *TI* = 100 ms, flip angle = 5°, FoV = 240 × 240 mm^2^, matrix = 240 × 240, 170 slices, voxel size = 1 × 1 × 1 mm^3^). FMRI data were obtained by using a gradient echo EPI sequence (*TE* = 35 ms, *TR* = 2000 ms, flip angle = 82°, FoV = 220 × 220 mm^2^, matrix = 80 × 80, 32 slices, slice thickness = 4 mm, and 0 mm interslice gap; 300 volumes).

### fMRI data analysis

#### Preprocessing

For rs-fMRI data, SPM8 (Wellcome Department of Cognitive Neurology, London) was used for motion correction, spatial normalization into the stereotactic space of the Montreal Neurological Institute (MNI) and spatial smoothing with a 6 × 6 × 6 mm Gaussian kernel. To control for potential differences in motion between groups and potential bias on function connectivity (Van Dijk et al., 2012), several parameters have been investigated and compared between patients with MDD and healthy controls as reported previously [see (Meng et al., 2013) for extensive presentation of the applied procedures and analyses]. Briefly, excessive head motion (cumulative translation > 3 mm and rotation >3° as well as mean point-to-point translation >0.15 mm or rotation >0.1°) has been applied as exclusion criteria for all participants. Furthermore, two-sample *t*-tests were performed to investigate potential between-group differences in cumulative and/or mean point-to-point motion, both yielding no significant between-group differences, respectively (*p* > 0.05). Moreover, signal-to-noise ratio of fMRI data was not different between healthy subjects and patient group (two-sample *t*-test, *p* > 0.05).

#### Independent component analysis

Independent Component Analysis (ICA) is a computational technique for identifying statistically independent sources from multivariate data and can therefore be used to explore functional connectivity patters in the context of resting-state fMRI (Beckmann, 2012). In contrast to seed-based approaches, ICA analyzes the data in a data-driven way (Calhoun et al., 2001). Therefore, no a-prior assumptions, such as the manual selection of regions-of-interest (ROIs), is necessary, making ICA a powerful tool to investigate the complete picture of the functional hierarchy within the human brain (Cole et al., 2010), which we aimed to investigate in patients with MDD and healthy controls in the present study. As recently proposed by Allen et al. (2011) and previously reported (Manoliu et al., 2013b), preprocessed data were decomposed into 75 spatial independent components within a group-ICA framework (Calhoun et al., 2001), based on the infomax-algorithm and implemented in the GIFT-software (http://icatb.sourceforge.net). High-model-order ICA approaches yield independent components, which are in accordance with known anatomical and functional segmentations (Allen et al., 2011). FMRI data were concatenated and reduced by two-step principal component analysis, followed by independent component estimation with the infomax-algorithm. We subsequently ran 20 ICA (ICASSO) to ensure stability of the estimated components. This results in a set of average group components, which are then back reconstructed into single subject space via GICA3, a back-reconstruction algorithm based on PCA compression and projection [see (Allen et al., 2011) for detailed discussion of advantages of the GICA3 algorithm]. Each back-reconstructed component consists of a spatial z-map reflecting component's functional connectivity pattern across space (intra-iFC) and an associated time course reflecting component's activity across time.

#### Selection of model-order and networks-of-interest

**Figure 1:**
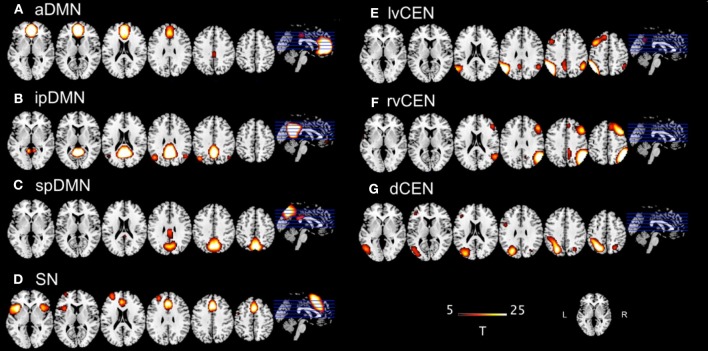
**T-maps of intrinsic connectivity networks of interest as described and provided online by Allen et al. (2011)**. Allen and colleagues used an ICA model-order of 75 to decompose rs-fMRI data of 603 subjects, obtaining 28 components. T-maps of components were provided online (http://mialab.mrn.org/data/hcp/RSN_HC_unthresholded_tmaps.nii). As previously reported (Manoliu et al., 2013b), we chose the T-maps of ICs representing the default mode network, salience network and central executive network, and performed multiple spatial regression analyses of our 75 independent components' spatial maps on these templates to select the networks-of-interest in an automated and objective way. Here, provided T-maps were superimposed on a single-subject high resolution T1 image (color scale representing *t*-values from 5 to 25). **(A)** Anterior default mode network (aDMN); **(B**) inferior posterior default mode network (ipDMN); **(C)** superior posterior default mode network (spDMN); **(D)** salience network (SN); **(E)** left ventral central executive network (lvCEN); **(F)** right ventral executive network (rvCEN); **(G)** dorsal central executive network (dCEN). Modified from Manoliu et al., 2013a.

Although ICA-based analyses of rs-fMRI data are often reported, the selection of the optimal ICA model-order to analyze rs-fMRI data is still a subject of ongoing debate [see Manoliu et al. (2013b) as well as Manoliu et al. (2013a) for detailed discussion]. However, it has been demonstrated that a model-order around 70 components may represent an optimal level to detect between-group differences and to avoid false positive results (Abou-Elseoud et al., 2010). Bearing this in mind and exactly following a recently proposed approach of Allen et al. (2011), we decomposed our data into 75 independent components. The congruence with Allen's approach enables greater comparability of results across studies and reduced subjective bias for ICN selection. In more detail, Allen and colleagues used an ICA model-order of 75 to decompose rs-fMRI data of 603 subjects within a group-ICA framework based on the infomax-algorithm and implemented in the GIFT-software (http://icatb.sourceforge.net) (Calhoun et al., 2001). Authors provided T-maps of 28 components, which reflect canonical ICNs online (http://mialab.mrn.org/data/hcp/RSN_HC_unthresholded_tmaps.nii; Allen et al., 2011). To select components, which reflect networks-of-interest, in an automated and objective way, we chose from these T-maps those representing subsystems of the SN, DMN, and CEN (7 of 28 maps, see Figure 1), and performed multiple spatial regression analyses of our 75 independent components' spatial maps on these templates. We selected components of highest correlation coefficient with the templates, resulting in 7 ICNs of interest: 1 component reflecting the SN, 3 reflecting subsystems of the DMN or CEN, respectively. In the end, this approach yielded for each subject and ICN a component's z-map and time course, which reflect network's coherent activity.

#### Outcome measures and statistical analysis

***Intra-iFC*.** To statistically evaluate intra-iFC of selected ICs, we calculated voxel-wise one-sample *t*-tests on participants' reconstructed spatial maps for each group, using SPM8 [*p* < 0.05, family-wise-error (FWE)-corrected for multiple comparisons]. To analyze group differences, participants' spatial maps were entered into two-sample *t*-tests with age, sex, and total gray matter (GM) volumes within the area covered by the 7 networks-of-interest [see section Voxel-Based Morphometry (VBM) Analysis for detailed presentation of calculation of total gray matter] as covariate-of-no-interest (*p* < 0.05 FWE-corrected).

***Inter-iFC*.** To statistically evaluate inter-iFC, subject specific ICN time courses (TCs) were detrended, despiked, filtered using a fifth-order Butterworth low-pass filter with a high frequency cutoff of 0.15 Hz, and pairwise correlated by Pearson's correlation, following the approach of Jafri et al. (2008) as reported in Manoliu et al. (2013b). To assess group differences, correlation coefficients were transformed to z-scores using Fisher's z-transformation and entered into two-sample *t*-tests with age, sex, and total GM volumes of the areas covered by the 7 networks-of-interest [see section Voxel-Based Morphometry (VBM) Analysis for details regarding the calculation of total gray matter] as covariate-of-no-interest (*p* < 0.05, Bonferroni-corrected for multiple comparisons).

***Correlation analyses*.** Insula dysfunction has been suggested to be associated with the severity of symptoms in patients with MDD (Menon, 2011; Hamilton et al., 2013). Accordingly, the total scores of both HAM-D and BDI were selected for further correlation analyses. To evaluate relationships between insula network connectivity (SN's insular intra-iFC) and both between-network interactions (inter-iFCs) and severity of symptoms in patients, we first calculated voxel-wise one-sample *t*-test on patients' reconstructed intra-iFC maps for the SN and masked the result with a mask derived from the two-sample-*t*-test contrasting patients from healthy controls. Subsequently, we extracted principle eigenvariates of left and right AI within patient's masked SN spatial map, respectively. Then we used eigenvariate-scores for partial correlation analyses of Fisher-z-transformed inter-iFC scores and both HAM-D scores and BDI scores, respectively, including age, sex, and total GM within the brain areas covered by the 7 networks-of-interest as covariates of no interest [see section Voxel-Based Morphometry (VBM) Analysis for detailed description of the calculation of total gray matter]. To study the relationship between inter-iFCs and severity of depressive symptoms in patients, we used Fisher-z-transformed inter-iFC scores for partial correlation analyses of both HAM-D scores and BDI scores, respectively, including age, sex, and total GM within the brain areas covered by the 7 networks-of-interest as covariates of no interest. Results of partial correlation analyses were thresholded at *p* < 0.05, Bonferroni-corrected for multiple comparisons.

### Voxel-based morphometry (VBM) analysis

The VBM analysis followed the description provided in Meng et al. (2013). The functional connectivity of intrinsic brain networks depends on widespread structural integrity of polysynaptic pathways (Lu et al., 2011). Since we focus on alterations of functional interactions among 7 distinct networks, we included total GM scores of the brain areas covered by the 7 networks-of-interest as covariate-of-no-interest in above-mentioned FC analyses to control for the influence of structural variations. As described recently (Sorg et al., 2013), we used the VBM8 toolbox (http://dbm.neuro.uni-jena.de/vbm.html) to analyze brain structure. T1-weighted images were corrected for bias-field inhomogeneity, registered using linear (12-parameter affine) and non-linear transformations, and tissue-classified into gray matter (GM), white matter (WM), and cerebro-spinal fluid (CSF) within the same generative model (Ashburner and Friston, 2005). The resulting GM images were modulated to account for volume changes resulting from the normalization process. Here, we only considered non-linear volume changes so that further analyses did not have to account for differences in head size. Finally images were smoothed with a Gaussian kernel of 8 mm (FWHM). Since we were interested specifically in investigating aberrant iFC within and between 7 distinct ICNs, we constructed a binarized mask representing all 7 ICNs of interest and extracted the total GM scores within this mask for each group following the procedure reported in Manoliu et al. (2013b). Subsequently, we used these scores as covariate of no interest in all further analyses of both intra-iFC and inter-iFC within and between the 7 ICNs of interest. Furthermore, between group comparisons have been reported in a previous study (Meng et al., 2013).

## Results

### Intrinsic connectivity networks: intra- and inter-iFC

**Figure 2:**
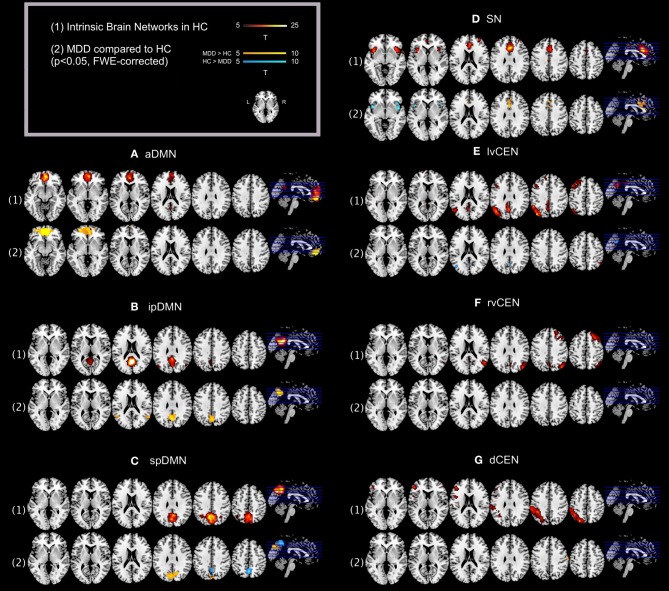
**Default mode network, salience network, and central executive network in healthy controls and corresponding group differences for patients with major depressive disorder**. (1) Spatial maps of selected ICs (derived from ICA of fMRI data) representing the default mode, salience, and central executive network (DMN, SN, CEN) in healthy controls were entered into voxel-wise one-sample *t*-tests and thresholded at *p* < 0.05, corrected for family wise error (FWE). Statistical parametric maps (SPMs) representing brain areas with significantly co-varying activity were superimposed on a single-subject high resolution T1 image (color scale representing *t*-values from 5 to 25; only maps of healthy controls are shown). (2) To analyze between-group differences, patients' and controls' ICs of the DMN, SN, and CEN were entered into voxel-wise two-sample-*t*-test with age, sex, and GM volume of the brain areas covered by the 7 networks of interest as covariates of no interest and thresholded at *p* < 0.05, FWE-corrected. SPMs were superimposed on a single-subject high resolution T1 image (color scale representing *t*-values from 5 to 10; yellow (“hot”) color maps indicate regions displaying higher intra-iFC in MDD compared to HC; blue (“cold”) color maps indicate regions displaying lower intra-iFC in MDD compared to HC). Results of between-group comparisons are presented panel-wise. **(A)** Anterior default mode network (aDMN); **(B)** inferior posterior default mode network (ipDMN); **(C)** superior posterior default mode network (spDMN); **(D)** salience network (SN); **(E)** left ventral central executive network (lvCEN); **(F)** right ventral executive network (rvCEN); **(G)** dorsal central executive network (dCEN). Abbreviations: MDD, group of patients with major depressive disorder; HC, healthy control group (see also Tables 2, 3).

**Figure 3:**
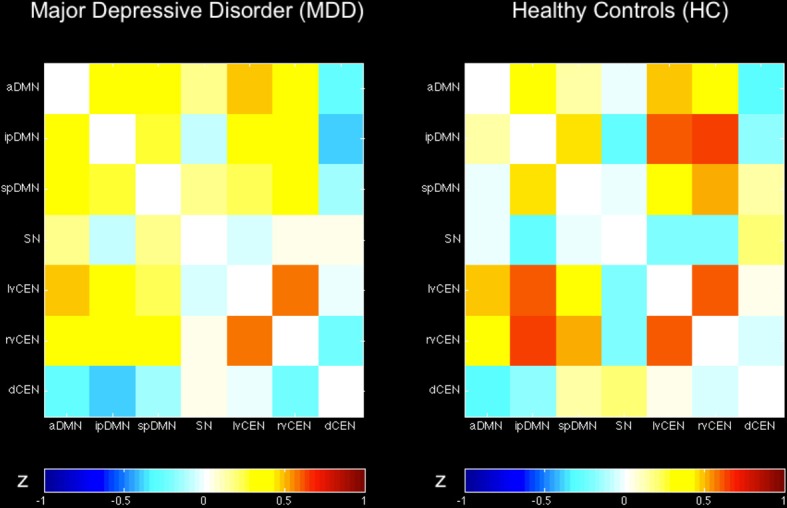
**Inter-network intrinsic functional connectivity matrix for patients with major depressive disorder and healthy controls**. Pairwise Pearson's correlations between time courses of selected ICs (derived from ICA of fMRI data) concerning the default mode, salience, and central executive network (DMN, SN, CEN) were Fisher-z-transformed, averaged across subjects for each group of patients with major depressive disorder and healthy controls, and presented in a correlation matrix. Colors represent intensity of averaged z-scores. a/ip/spDMN, anterior/inferior-posterior/superior-posterior DMN; SN, salience network; lv/rv/dCEN, left-ventral/right-ventral/dorsal CEN (see Table 4).

Regarding both intra-iFC and inter-iFC, current results matched almost perfectly reported findings of Allen et al. (2011) and previously reported studies investigating the iFC within and between the DMN, SN, and CEN (Manoliu et al., 2013b), demonstrating the presence of the basic functional architecture of the DMN, SN, and CEN in both investigated groups (see Figure 1 for presentation of spatial templates, Figure 2 and Table 2 for detailed presentation of intra-iFC within ICNs of interest and Figure 3, and Table 4 for detailed presentation of inter-iFC between ICNs of interest).

**Table 2:** **Intrinsic connectivity networks in healthy controls**.

**Anatomical region**	**L/R/Bi**	**Cluster**	***Z*-score**	*p*-value^*^	**MNI^a^(*x*, *y*, *z*)**
**(A) ANTERIOR DEFAULT MODE NETWORK (aDMN)**					
Medial prefrontal cortex	L	1224	>8.00	<0.001	0, 50, −14
Medial prefrontal cortex	R	”	>8.00	<0.001	6, 47, −5
Posterior cingulate cortex	Bi	47	6.14	<0.001	0, −55, 19
**(B) INFERIOR POSTERIOR DEFAULT MODE NETWORK (ipDMN)**					
Precuneus	L	937	>8.00	<0.001	−3, −52, 22
Precuneus	R	”	>8.00	<0.001	3, −55, 19
Angular gyrus	L	40	7.33	<0.001	−45, −73, 37
Angular gyrus	R	17	6.06	<0.001	48, −67, 31
**(C) SUPERIOR POSTERIOR DEFAULT MODE NETWORK (spDMN)**					
Precuneus	R	1444	>8.00	<0.001	6, −64, 43
Precuneus	L	”	>8.00	<0.001	0, −79, 43
Angular gyrus	L	”	6.20	<0.001	−36, −58, 40
Angular gyrus	R	56	6.15	<0.001	36, −58, 37
**(D) SALIENCE NETWORK (SN)**					
Anterior cingulate cortex	Bi	948	>8.00	<0.001	0, 23, 28
Anterior insula	L	432	>8.00	<0.001	−39, 14, −11
Anterior insula	R	400	>8.00	<0.001	51, 17, −11
Middle frontal gyrus	R	92	6.52	<0.001	33, 53, 22
**(E) LEFT VENTRAL CENTRAL EXECUTIVE NETWORK (lvCEN)**					
Angular gyrus	L	653	>8.00	<0.001	−45, −67, 31
Inferior parietal gyrus	L	”	7.82	<0.001	−51, −52, 31
Precuneus	L	310	7.60	<0.001	−3, −61, 31
Middle frontal gyrus	L	345	6.73	<0.001	−45, 17, 49
Inferior temporal gyrus	L	24	6.27	<0.001	−63, −49, −17
Superior medial gyrus	L	33	5.55	<0.001	−9, 41, 40
**(F) RIGHT VENTRAL CENTRAL EXECUTIVE NETWORK (rvCEN)**					
Superior frontal gyrus	R	481	7.46	<0.001	30, 26, 52
Angular gyrus	R	488	7.40	<0.001	42, −73, 40
Precuneus	R	94	6.68	<0.001	3, −61, 25
Middle frontal gyrus	L	25	6.49	<0.001	−24, 17, 58
Temporal pole	L	10	5.71	<0.001	−57, 11, −11
**(G) DORSAL CENTRAL EXECUTIVE NETWORK (dCEN)**					
Supramarginal gyrus	L	1099	7.79	<0.001	−60, −34, 37
Inferior frontal gyrus	L	182	7.22	<0.001	−45, 38, 7
Inferior frontal gyrus	L	109	7.04	<0.001	−48, 8, 22
Inferior temporal gyrus	L	161	6.75	<0.001	−57, −55, −11
Superior frontal gyrus	L	35	6.31	<0.001	−24, 5, 64
Middle frontal gyrus	R	9	5.84	<0.001	51, 44, 10

* One-sample-t-test, significant for p < 0.05, FWE-corrected for multiple comparisons, cluster-threshold >10 voxel.

a MNI, Montreal Neurological institute; L, left hemisphere; R, right hemisphere; Bi, bilateral (see Figure 2).

#### Intra-iFC

As previously described (Manoliu et al., 2013b), automated component selection, which was based on spatial templates representing subsystems of the DMN, SN, and CEN, revealed 7 components of interest for each participant [see Figure 1 for presentation of spatial templates according to Allen et al. (2011)]: The SN was represented in one component (SN, corresponding with Allen-IC 55). The DMN was represented in 3 components [anterior DMN (aDMN, corresponding with Allen-IC 25), inferior posterior DMN (ipDMN, corresponding with Allen-IC 53), superior posterior DMN (spDMN, corresponding to Allen-IC 50)]. The CEN was represented in 3 components [left ventral CEN (lvCEN, corresponding to Allen IC 34), right ventral CEN (rvCEN, corresponding to Allen-IC 60), dorsal CEN (dCEN, corresponding to Allen-IC 52)]. All selected components were spatially consistent across groups and matched previous results of SN, DMN, and CEN (Allen et al., 2011) (see Figure 2 and Table 3 for detailed description of intra-iFC within selected ICNs, *p* < 0.05, FWE-corrected).

**Table 3:** **Altered intra-iFC in patients with major depressive disorder compared to healthy controls**.

**Anatomical region**	**L/R/Bi**	**Cluster**	***Z*-score**	*p*-value^*^	**MNI^a^ (*x*, *y*, *z*)**
**(A) ANTERIOR DEFAULT MODE NETWORK (aDMN)**					
**(a) MDD > HC**					
Anterior cingulate cortex	R	635	>8.00	<0.001	6, 47, −14
Anterior cingulate cortex	L	”	>8.00	<0.001	0, 65, 4
**(b) MDD < HC**					
–					
**(B)INFERIOR POSTERIOR DEFAULT MODE NETWORK (ipDMN)**					
**(a) MDD > HC**					
Precuneus	L	292	>8.00	<0.001	0, −64, 28
Precuneus	R	”	6.25	<0.001	9, −52, 31
**(b) MDD < HC**					
–					
**(C) SUPERIOR POSTERIOR DEFAULT MODE NETWORK (spDMN)**					
**(a) MDD > HC**					
Precuneus	R	275	6.91	<0.001	15, −64, 28
Precuneus	L	”	6.77	<0.001	−12, −70, 25
**(b) MDD < HC**					
Precuneus	R	157	7.14	<0.001	3, −61, 46
Precuneus	L	13	5.95	<0.001	−6, −49, 58
**(D) SALIENCE NETWORK (SN)**					
**(a) MDD > HC**					
Anterior cingulate cortex	Bi	168	6.54	<0.001	0, 8, 34
**(b) MDD < HC**					
Insula lobe	R	65	5.05	0.007	48, 14, −2
Insula lobe	L	53	4.91	0.017	−42, 11, −8
**(E) LEFT VENTRAL CENTRAL EXECUTIVE NETWORK (lvCEN)**					
**(a) MDD > HC**					
Angular gyrus	R	23	5.34	0.002	51, −61, 46
**(b) MDD < HC**					
Precuneus	L	26	5.52	<0.001	0, −64, 25
Middle temporal gyrus	L	24	5.48	<0.001	−42, −70, 19
**(F) RIGHT VENTRAL CENTRAL EXECUTIVE NETWORK (rvCEN)**					
**(a) MDD > HC**					
–					
**(b) MDD < HC**					
–					
**(G) DORSAL CENTRAL EXECUTIVE NETWORK (dCEN)**					
**(a) MDD > HC**					
Postcentral gyrus	R	19	5.58	<0.001	63, −16, 40
**(b) MDD < HC**					
–					

* Two-sample-t-test with age, sex, and total GM volume within brain areas covered by the 7 ICNs of interest as covariates of no-interest, significant for p < 0.05, FWE-corrected for multiple comparisons. cluster-threshold > 10 voxel.

a MNI, Montreal Neurological institute; L, left hemisphere; R, right hemisphere, Bi, bilateral (see Figure 2).

#### Inter-iFC

Calculated inter-iFC between ICNs of interest matched results of Allen et al. (2011), (see Figure 3 and Table 4 for detailed presentation of inter-iFC between all ICNs of interest). In accordance with previous findings (Manoliu et al., 2013b), we found positive correlations between subsystems of the DMN and CEN in both groups. Despite the incongruity with previously described patterns of anti-correlation between the DMN and CEN (Fox and Raichle, 2007), it is well in line with findings presented in recent studies using high-model order ICA (Allen et al., 2011). In particular, Smith et al. (2012) demonstrated by applying high temporal resolution resting-state fMRI that distinct sub-networks within the DMN are associated with distinctive pattern of between-network connectivity, possibly underlying the constantly shown heterogeneous connectivity pattern between subsystems of the DMN and CEN.

**Table 4:** **Inter-network intrinsic functional connectivity in patients with major depressive disorder and healthy controls**.

**Inter-iFC**	**MDD (***n*** = 25**)		**HC (***n*** = 25**)		**MDD vs. HC^a^**	
	**Mean**	***SD***	**Mean**	***SD***	**Direction**	***p*-value**
aDMN—ipDMN	0.412	0.281	0.313	0.250	MDD > HC	0.088
aDMN—spDMN	0.340	0.247	0.131	0.244	MDD > HC	*0.003*
aDMN—SN	0.183	0.223	−0.020	0.248	MDD > HC	*0.029*
aDMN—lvCEN	0.485	0.250	0.494	0.209	HC > MDD	0.684
aDMN—rvCEN	0.410	0.231	0.380	0.191	MDD > HC	0.896
aDMN—dCEN	−0.266	0.233	−0.294	0.184	MDD > HC	0.593
ipDMN—spDMN	0.274	0.221	0.466	0.189	HC > MDD	0.062
ipDMN—SN	−0.070	0.185	−0.276	0.224	MDD > HC	0.002^*^
ipDMN—lvCEN	0.383	0.245	0.616	0.203	HC > MDD	*0.005*
ipDMN—rvCEN	0.427	0.208	0.629	0.205	HC > MDD	*0.019*
ipDMN—dCEN	−0.393	0.235	−0.162	0.184	HC > MDD	0.001^*^
spDMN—SN	0.160	0.202	−0.018	0.261	MDD > HC	0.054
spDMN—lvCEN	0.244	0.243	0.431	0.215	HC > MDD	0.080
spDMN—rvCEN	0.395	0.193	0.509	0.247	HC > MDD	0.438
spDMN—dCEN	−0.134	0.208	0.131	0.217	HC > MDD	<0.001^*^
SN—lvCEN	−0.044	0.173	−0.209	0.217	MDD > HC	*0.027*
SN—rvCEN	0.048	0.216	−0.194	0.277	MDD > HC	*0.021*
SN—dCEN	0.043	0.217	0.201	0.204	HC > MDD	*0.026*
lvCEN—rvCEN	0.580	0.248	0.602	0.202	HC > MDD	0.901
lvCEN—dCEN	−0.030	0.248	0.056	0.199	HC > MDD	0.120
rvCEN—dCEN	−0.230	0.222	−0.055	0.245	HC > MDD	*0.021*

a Two-sample t-test controlled for age, sex and total GM volume within brain areas covered by the 7 ICNs of interest.

Italics indicate p < 0.05;

* significant for p < 0.05, Bonferroni-corrected for multiple comparisons (n = 21).

**Figure 4:**
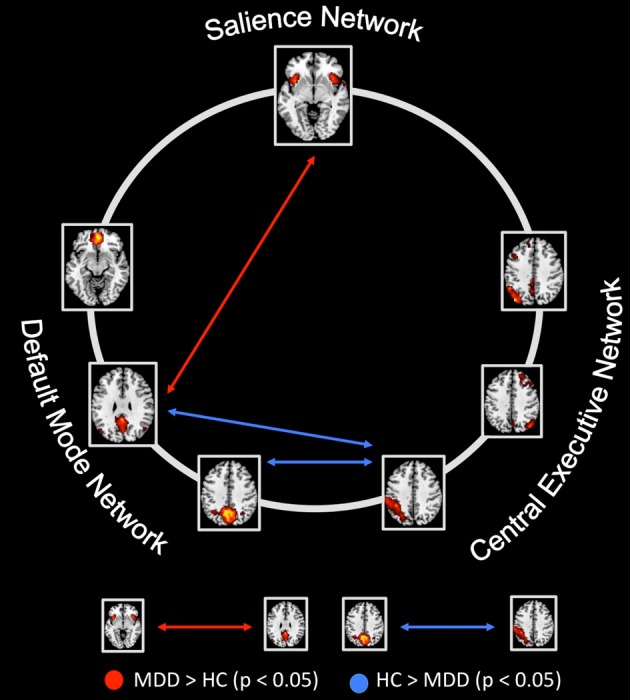
**Between-group differences of inter-network intrinsic functional connectivity**. Based on network time courses, inter-network intrinsic functional connectivity (inter-iFC) was calculated by the use of Pearson's correlation between subject specific ICN timecourses (TCs). The red arrows indicates increased inter-iFC in patients with major depressive disorder compared to healthy controls (two-sample *t*-test, *p* < 0.05, Bonferroni-corrected for multiple comparisons); The blue arrows indicates decreased inter-iFC in patients with major depressive disorder compared to healthy controls (two-sample *t*-test, *p* < 0.05, Bonferroni-corrected for multiple comparisons). Spatial maps indicate the anterior/inferior-posterior/superior-posterior default mode network (a/ip/spDMN), left-ventral/right-ventral/dorsal central executive network (lv/rv/dCEN), and salience network (SN). All tests were corrected for age, sex, and GM volume of the brain areas covered by the 7 networks of interest. Abbreviations: MDD, group of patients with major depressive disorder; HC, healthy control group (see also Table 4).

Abbreviations: MDD, group of patients with major depressive disorder; HC, healthy control group; inter-iFC, inter-network intrinsic functional connectivity; a/ip/spDMN, anterior/inferior-posterior/superior-posterior DMN; lv/rv/dCEN, left-ventral/right-ventral/dorsal CEN; SN, salience network (see also Figures 3, 4).

### Intra-iFC of the SN is disrupted in bilateral anterior insula in patients with major depressive disorder

Compared to healthy controls, patients demonstrated altered intra-iFC within the DMN, SN, and CEN. (Figure 2 and Table 3; *p* < 0.05 FWE-corrected with age, sex, and total GM of the brain areas covered by the 7 networks of interest as covariates of no-interest). Regarding the SN, patients showed decreased intra-iFC within the bilateral AI. Furthermore, intra-iFC was increased in bilateral ACC within the SN (see Figure 2D). Regarding the DMN, patients showed increased intra-iFC in bilateral ACC within the aDMN (see Figure 2A), increased intra-iFC within the in the bilateral precuneus within the ipDMN (see Figure 2B) as well as both increased and decreased intra-iFC in distinct parts of the precuneus within the spDMN (see Figure 2C). Regarding the CEN, patients showed heterogeneous alterations, including increased intra-iFC in the right angular gyrus and decreased intra-iFC in both the left precuneus and left middle temporal gyrus within the lvCEN (see Figure 2E) as well as increased intra-iFC in the right postcentral gyrus within the dCEN (see Figure 2G). No between-group differences were observed within the rvCEN (see Figure 2F).

### Inter-iFC between DMN and CEN is decreased in patients with major depressive disorder

Compared to healthy controls, patients with major depressive disorder showed both increased and decreased inter-iFC (see Figure 4, Table 4; *p* < 0.05, corrected for age, sex and GM volume of the brain areas covered by the 7 networks of interest, Bonferroni-corrected for multiple comparisons). Patients showed decreased inter-iFC between ipDMN and dCEN as well as between spDMN and dCEN, suggesting a decreased functional connectivity between the DMN and CEN. Furthermore, patients showed increased inter-iFC between SN and ipDMN, indicating increased functional connectivity between the SN and DMN.

### Right anterior insula's aberrant SN connectivity is associated with severity of symptoms in patients with major depressive disorder

**Figure 5:**
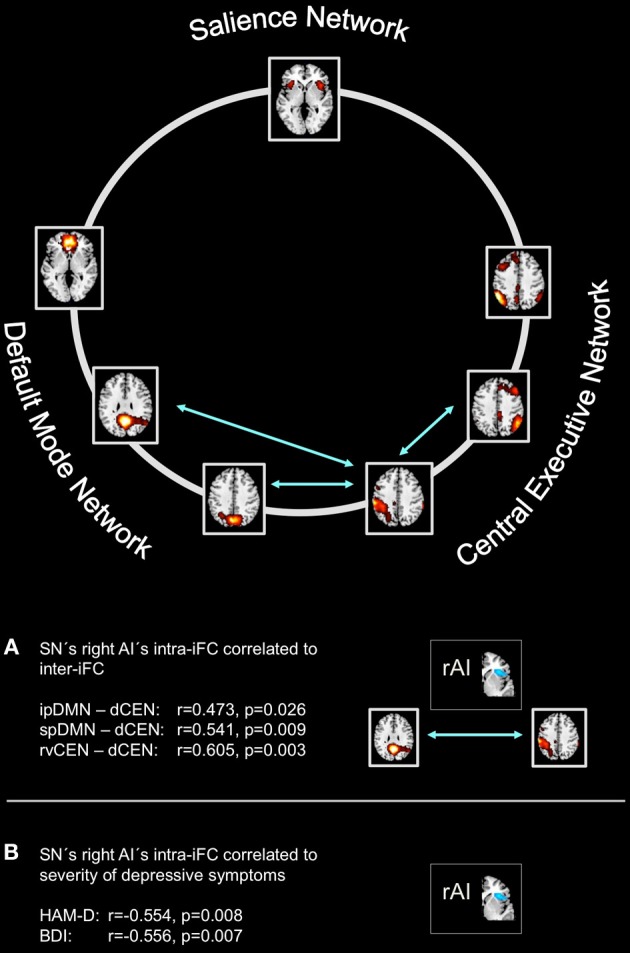
**Intra-iFC in the right anterior insula within the salience network is associated with severity of symptoms and inter-iFC between DMN and CEN in patients with major depressive disorder**. Intrinsic functional connectivity (inter-iFC) between ICNs of interest was calculated by the use of Pearson's correlation between networks' time courses. **(A)** Intra-iFC in the right anterior insula within the SN (turquoise spatial map) was significantly correlated with severity of negative symptoms in patients with major depressive disorder as measured by both HAM-D (partial correlation, *r* = −0.554, *p* = 0.008) and BDI (partial correlation, *r* = −0.556, *p* = 0.007). **(B)** Intra-iFC in the right anterior insula within the SN was significantly correlated with the inter-iFC between DMN and CEN in patients (turquoise arrows, partial correlations, ipDMN—dCEN: *r* = 0.472, *p* = 0.026; spDMN—dCEN: *r* = 0.541, *p* = 0.009). Furthermore, intra-iFC in the right anterior insula within the SN was significantly correlated with the inter-iFC between distinct subsystems of the CEN (rvCEN—dCEN: *r* = 0.605, *p* = 0.003). All partial correlations were corrected for age, sex, and GM volume of the brain areas covered by the 7 networks of interest. Spatial maps indicate the anterior/inferior-posterior/superior-posterior default mode network (a/ip/spDMN), left-ventral/right-ventral/dorsal central executive network (lv/rv/dCEN), and salience network (SN) (see also Tables 5–7).

To study the influence of insular SN activity on the severity of symptoms in patients with MDD, we correlated eigenvariates of SN's left and right AI group difference clusters with both HAM-D and BDI total scores (Figure 5, Table 5; *p* < 0.05, partial correlations with age, sex, and GM of the brain areas covered by the 7 networks of interest as covariates of no-interest, Bonferroni-corrected for multiple comparisons). In patients, SN's right AI's intra-iFC correlated negatively with the severity of symptoms as measured by both HAM-D (*r* = −0.554, *p* = 0.008) and BDI (*r* = −0.556, *p* = 0.007), suggesting an association between aberrant connectivity within the right anterior insular cortex and the severity of symptoms in patients with MDD. The altered intra-iFC within the left AI did not show any significant correlation with total scores assessed by HAM-D and BDI, respectively.

**Table 5:** **Partial correlations between intra-iFC in the right/left AI within the SN and severity of symptoms in patients with major depressive disorder**.

	**Right AI**		**Left AI**	
	***r*-score**	***p*-value**	***r*-score**	***p*-value**
HAM-D	−0.554	0.008^*^	0.103	0.649
BDI	−0.556	0.007^*^	−0.320	0.888

Italics indicate p < 0.05;

* significant for p < 0.05, after Bonferroni-correction for multiple comparisons (n = 2).

Partial correlation, corrected for age, sex and total GM volume within brain areas covered by the 7 ICNs of interest.

Abbreviations: AI, anterior Insula; HAM-D, Hamilton Depression Rating Scale; BDI, Beck Depression Inventory (see Figure 5).

### Right anterior insula's aberrant SN connectivity is associated with altered DMN-CEN interaction in patients with major depressive disorder

To study the influence of insular SN activity on altered inter-network connectivity in patients, we correlated eigenvariates of SN's left and right AI group difference clusters with Fisher-z-transformed correlation coefficients of each pair of network time courses (Figure 5, Table 6, *p* < 0.05, partial correlations with age, sex, and GM of the brain areas covered by the 7 networks of interest as covariates of no-interest, Bonferroni-corrected for multiple comparisons). In patients, SN's right AI's intra-iFC correlated positively with inter-iFC between ipDMN and dCEN (*r* = 0.472, *p* = 0.026) as well as between spDMN and dCEN (spDMN—dCEN: *r* = 0.541, *p* = 0.009). Furthermore, the SN's right AI's intra-iFC correlated positively with the aberrant inter-iFC between the rvCEN and dCEN (*r* = 0.605, *p* = 0.003). These results suggest a relation between altered connectivity within the right anterior insular cortex and aberrant inter-network connectivity in patients with MDD. However, it is to note that these associations were not significant when corrected for multiple comparisons (*n* = 21). Furthermore, the altered intra-iFC in the left AI within the SN did not show any significant correlation to inter-network connectivity.

**Table 6:** **Partial correlations between intra-iFC in the right/left AI within the SN and inter-iFC in patients with major depressive disorder**.

**Inter-iFC**	**Right AI**		**Left AI**	
	***r*-score**	***p*-value**	***r*-score**	***p*-value**
aDMN—ipDMN	−0.24	0.282	−0.23	0.304
aDMN—spDMN	−0.195	0.383	0.108	0.633
aDMN—SN	0.285	0.198	−0.204	0.362
aDMN—lvCEN	0.243	0.277	−0.001	0.998
aDMN—rvCEN	−0.3	0.174	−0.043	0.851
aDMN—dCEN	0.412	0.056	0.135	0.548
ipDMN—spDMN	−0.24	0.282	0.118	0.602
ipDMN—SN	0.087	0.702	0.059	0.793
ipDMN—lvCEN	0.11	0.627	−0.325	0.14
ipDMN—rvCEN	−0.403	0.063	−0.051	0.823
ipDMN—dCEN	0.473	*0.026*	0.453	*0.034*
spDMN—SN	0.33	0.134	−0.201	0.369
spDMN—lvCEN	−0.23	0.302	0.205	0.359
spDMN—rvCEN	−0.403	0.063	0.318	0.15
spDMN—dCEN	0.541	*0.009*	−0.057	0.8
SN—lvCEN	0.249	0.263	−0.117	0.605
SN—rvCEN	0.416	0.054	−0.118	0.601
SN—dCEN	−0.045	0.843	−0.119	0.598
lvCEN—rvCEN	−0.266	0.232	0.186	0.406
lvCEN—dCEN	0.097	0.669	−0.147	0.514
rvCEN—dCEN	0.605	*0.003*	0.019	0.932

Italics indicate p < 0.05.

Partial correlation, corrected for age, sex and total GM volume within brain areas covered by the 7 ICNs of interest.

Abbreviations: a/ip/spDMN, anterior/inferior posterior/superior posterior DMN; lv/rv/dCEN, left ventral/right ventral/dorsal CEN; SN, salience network; AI, anterior insula (see Figure 5).

### Altered inter-network connectivity was not associated with the severity of symptoms

To study the relationship between inter-network connectivity and severity of symptoms, we correlated inter-iFC scores with total scores as assessed with both HAM-D and BDI, respectively (Figure 5, Table 7; *p* < 0.05, partial correlations with age, sex and GM of the brain areas covered by the 7 networks of interest as covariates of no-interest, Bonferroni-corrected for multiple comparisons). There was no association between inter-network connectivity and severity of symptoms in patients with major depressive disorder.

**Table 7:** **Partial correlations between inter-iFC and severity of symptoms in patients with major depressive disorder**.

**Inter-iFC**	**HAM-D**		**BDI**	
	***r*-score**	***p*-value**	***r*-score**	***p*-value**
aDMN—ipDMN	0.076	0.735	0.082	0.716
aDMN—spDMN	0.098	0.664	0.009	0.969
aDMN—SN	−0.048	0.833	−0.214	0.34
aDMN—lvCEN	0.029	0.896	0.195	0.386
aDMN—rvCEN	0.398	0.067	0.429	*0.047*
aDMN—dCEN	0.054	0.811	−0.057	0.8
ipDMN—spDMN	−0.024	0.916	0.058	0.797
ipDMN—SN	−0.055	0.808	−0.282	0.203
ipDMN—lvCEN	−0.155	0.49	−0.116	0.609
ipDMN—rvCEN	0.305	0.167	0.199	0.375
ipDMN—dCEN	0.059	0.794	−0.119	0.597
spDMN—SN	0.039	0.864	−0.114	0.614
spDMN—lvCEN	0.128	0.57	−0.008	0.971
spDMN—rvCEN	0.178	0.429	0.203	0.364
spDMN—dCEN	0.11	0.626	−0.041	0.856
SN—lvCEN	−0.107	0.637	−0.243	0.277
SN—rvCEN	−0.306	0.167	−0.371	0.089
SN—dCEN	0.101	0.656	0.004	0.987
lvCEN—rvCEN	0.504	*0.017*	0.353	0.108
lvCEN—dCEN	−0.109	0.629	−0.142	0.527
rvCEN—dCEN	−0.289	0.193	−0.315	0.153

Italics indicate p < 0.05.

Partial correlation, corrected for age, sex and GM volume within brain areas covered by the 7 ICNs of interest.

Abbreviations: HAM-D, Hamilton Depression Rating Scale; BDI, Beck Depression Inventory; a/ip/spDMN, anterior/inferior posterior/superior posterior DMN; lv/rv/dCEN, left ventral/right ventral/dorsal CEN; SN, salience network.

## Discussion

To investigate the relationship between anterior insular dysfunction within the SN, altered between-network interaction, and severity of symptoms in MDD, we analyzed intrinsic functional connectivity within (intra-iFC) and between (inter-iFC) the DMN, SN, and CEN by the use of resting-state fMRI and ICA in patients with MDD and healthy controls. We found aberrant intra-iFC in DMN, SN and CEN, including decreased intra-iFC in the right AI within the SN in patients. Furthermore, we found decreased inter-iFC between subsystems of the DMN and CEN as well as increased inter-iFC between the SN and DMN. Remarkably, decreased intra-iFC in the right AI within the SN correlated significantly with the severity of symptoms. Furthermore, we found a correlation between the decreased intra-iFC in the SN's rAI and aberrant inter-iFC between subsystems of the DMN and CEN in patients with major depressive disorder but significance did not survive correction for multiple comparisons. These results extend our knowledge about aberrant iFC within intrinsic networks in MDD by revealing altered iFC across networks, more specifically the link between right AI dysfunction within the SN, aberrant between-network interaction and severity of symptoms in patients with MDD. Together with previously reported findings (Hamilton et al., 2011) and in accordance with recently suggested models (Menon, 2011; Hamilton et al., 2013), our data suggest aberrant insular control of DMN-CEN interactions, potentially contributing to depressive negative bias in attention and thought in MDD.

### The link between anterior insular dysfunction within the SN and aberrant interactions between DMN and CEN in MDD

In bilateral anterior insula, intra-iFC was decreased within the SN, while SN's inter-iFC with the ipDMN was increased. Furthermore, we found decreased inter-iFC between the ipDMN and dCEN, indicating both insular dysfunction and altered connectivity between subsystems of the DMN and CEN in patients with MDD. In addition, rAI's decreased intra-iFC correlated both negatively with the severity of symptoms, suggesting a link between insular dysfunction and severity of symptoms in major depression, and positively with the decreased connectivity between ipDMN and dCEN, suggesting a link between rAI dysfunction and aberrant DMN/CEN interactions. However, it is to note that the latter correlation lost significance after correction for multiple comparisons (*n* = 21). All these results were controlled for effects of age, sex and total GM of the brain areas covered by the networks of interest. Thus, it is unlikely that these possible confounders explain the reported results.

These findings support the hypothesis that rAI dysfunction might be associated with abnormal interactions between DMN and CEN in MDD, likely via impaired AI-mediated control of network interactions (Menon, 2011). Previous findings support this assumption: (1) The rAI has been demonstrated to play a pivot role in modulating interactions between DMN and CEN in healthy controls (Sridharan et al., 2008) and to show aberrant activity at the onset of increases in DMN and CEN activity, while aberrant relationship between the DMN and CEN was associated with severity of rumination in patients with MDD (Hamilton et al., 2011). Moreover, a recent meta-analysis performed by Diener et al. (2012) demonstrated, that the right AI consistently showed hypoactivity during affective switching and cognitive control tasks in MDD patients. (2) In patients with MDD, functional anomalies within the AI rank among the most frequently reported findings in the current literature (Diener et al., 2012; Sliz and Hayley, 2012; Hamilton et al., 2013). (3) More generally, recently formulated models propose that insular dysfunction and its consecutive abnormal modulation of interactions between networks (i.e., aberrant engagement and disengagement of the DMN and CEN due to aberrant AI mediated network switching) contribute to several neuropsychiatric disorders via aberrant mapping and detection of salient external stimuli and internal events (Menon and Uddin, 2010), potentially manifesting in specific symptom dimensions by specific AI/SN changes (Menon, 2011; Uddin et al., 2011; Palaniyappan and Liddle, 2012). For example we found recently that aberrant intra-iFC within the rAI is related to aberrant interaction between DMN and CEN in psychotic patients with schizophrenia (Manoliu et al., 2013b). Taken together, our data suggest a direct association between insular dysfunction, severity of symptoms and aberrant inter-network interactions via impaired insular control in patients with MDD.

Two further comments concerning laterality and specificity of our results might be useful: (i) Laterality: It is to note that although both right and left AI showed decreased intra-iFC within the SN, only patients' right AI showed significant correlation with the severity of symptoms while the left AI displayed no results. Furthermore, the decreased intra-iFC in the right AI within the SN correlated with the decreased connectivity between the DMN and CEN, whereas it is to note, however, that this correlation lost significance after performing correction for multiple comparisons (*n* = 21). This finding is consistent with previous observations: in healthy controls, the right AI modulates selectively inter-network interactions (Sridharan et al., 2008), in patients with MDD only the right AI is characterized by aberrant activity at the onset of DMN/CEN activity (Hamilton et al., 2011). This insular asymmetry might relate with the asymmetric representation of afferent sympathetic nervous system activity in the insula and the fact that interoceptive feelings are predominantly associated with the right AI (Craig, 2002, 2009). Therefore, our data might indicate a potential link between aberrant rAI control processes, sympathetic activity and interoception in patients with MDD. (ii) Specificity: Since increasing evidence for the relevance of right AI dysfunction is emerging in various neuropsychiatric disorders, Menon (2011) suggested, that anomalies within the right AI might contribute to aberrant inter-network interactions, leading to various symptom dimensions such as affective and psychotic symptoms via distinct disease-specific pathways. It is evident that presented results support this notion. However, it is still unclear how the triple network model of anomalies among DMN, SN, and CEN might be linked to anomalies beyond these networks (Williamson and Allman, 2012), such as aberrant reinforcement prediction error (Gradin et al., 2011), striatal reward processing (Robinson et al., 2012) and connectivity (Meng et al., 2013), or aberrant medial temporal lobe activity and connectivity (Tahmasian et al., 2013) in patients with MDD. Further studies investigating the possible link between aberrant DMN/SN/CEN organization and subcortical functional and/or neurochemical (Tahmasian et al., 2013) alterations in patients with MDD are necessary to better understand both the pathophysiology of MDD in particular and the nature of AI's involvement in psychiatric disorders in general.

### DMN/CEN interactions in patients with MDD

#### Intra-iFC within the DMN in patients with MDD

In patients with MDD, we found selectively increased intra-iFC within both aDMN and ipDMN as well as mainly increased intra-iFC within the spDMN. Furthermore, we found a trend to increased connectivity between aDMN and spDMN (*p* = 0.003) that, however, lost significance after correction for multiple comparisons (*n* = 21). Our findings are well in line with current literature reporting predominantly increased FC within the DMN during rest in patients with MDD (Greicius et al., 2007; Broyd et al., 2009; Posner et al., 2013). In particular, increased intra-iFC in the subgenual anterior cingulate and ventromedial prefrontal cortex of the DMN seems to be a highly robust finding in patients with depression (Greicius et al., 2007; Horn et al., 2010; Sheline et al., 2010; Veer et al., 2010). Considering that patients with MDD display less activation of the DMN in response to both positive and negative external stimuli [see Hamilton et al. (2013) for review], it has been proposed that the pattern of increased connectivity during rest and decreased activation during task might indicate that self-related cognition might be more susceptible to internal generated thoughts than to external stimuli in patients with MDD (Hamilton et al., 2013). Taken together, our results confirm previously reported findings, extending them by demonstrating that distinct subsystems of the DMN are consistently characterized by abnormal intra-iFC in patients with MDD.

#### Intra-iFC within the CEN in patients with MDD

In the current study, we found heterogeneous alterations in intra-iFC within the three sub-components of the CEN, including both increased intra-iFC in the right angular gyrus and decreased intra-iFC in the middle temporal gyrus and precuneus within the rvCEN. These findings are well in line with previous studies, which reported aberrant activity within right angular gyrus, middle temporal gyrus and precuneus in patients with MDD (Fitzgerald et al., 2008b). In general, the CEN, which is involved in goal-directed cognitively demanding tasks and control of emotional responses, has been shown to be altered in several psychiatric disorders, including MDD (Fitzgerald et al., 2008a; Pizzagalli et al., 2009; Menon, 2011; Diener et al., 2012). Although the CEN comprises both frontal and parietal regions, most studies investigated primarily the DLPFC, mainly due to the suggested link between functional anomalies within the DLPFC and impaired cognitive emotion regulation in MDD (Fitzgerald et al., 2008b). Several studies found heterogeneous results regarding both direction of effect (Diener et al., 2012) and exact localization (Hamilton et al., 2013) of aberrant iFC, possibly indicating that different findings might be located in different sub-networks of the CEN, each maintaining distinct tonic (resting-state) or phasic (affective response) cognitive processes (Hamilton et al., 2013). Our results confirm previous findings and support this notion that heterogeneously distributed anomalies of iFC are present within distinct subsystems of the CEN in MDD.

#### Inter-iFC between the DMN and CEN in patients with MDD

In the current study, we found decreased inter-iFC between the ipDMN and dCEN as well as between the spDMN and dCEN, indicating a decreased connectivity between DMN and CEN in patients with MDD. These results are well in line with previous findings (Sheline et al., 2010; Alexopoulos et al., 2013). Particularly Hamilton and colleagues found that increased dominance of the DMN was related to the severity of ruminations. It is to note that whereas the significant between-group differences regarding the inter-iFC between the ipDMN and dCEN are based on differences in height of negative correlations between the ipDMN and dCEN in both groups (see also Figures 3, 4 and Table 4), the group differences regarding the inter-iFC between spDMN and dCEN are based on the fact that timecourses between spDMN and dCEN are negatively correlated in patients with MDD and positively correlated in healthy controls. However, positive correlation between spDMN and dCEN in healthy controls corresponds with previous results (Allen et al., 2011) and has also been found in an independent group of healthy controls (see Manoliu et al., 2013b) using the same methodological approach as presented in the current study. As discussed previously (Manoliu et al., 2013a) positive correlation between subsystems of the DMN and CEN seems unexpected and in contrast to the notion of anti-correlation between DMN and CEN (Fox and Raichle, 2007). However, studies using high model order ICA (e.g., Allen et al., 2011) demonstrated that both DMN and CEN consist of functional sub-networks, while Smith et al. (2012) demonstrated by applying high temporal resolution resting-state fMRI that distinct sub-networks within the DMN are characterized by specific connectivity patterns between themselves and other networks. The current data suggests a partial re-organization of these inter-iFC patterns, particularly between subsystems of the DMN and CEN in patients with MDD.

Taken together, our findings replicate previously reported results about aberrant network connectivity in MDD, demonstrating the representative nature of our study sample. Moreover, we extend the current knowledge by linking aberrant intra-iFC in the AI within the SN with both aberrant inter-iFC between the DMN and CEN and severity of symptoms in patients with MDD.

### Psychopathological implications of altered large-scale brain networks in MDD

According to Beck's cognitive theory of depression (Beck, 1967; Beck and Alford, 2009), negative cognitive biases lead to a generally negative view of oneself and the world, thus underlying depression (Mathews and MacLeod, 2005; Willner et al., 2013). In particular, activation of negative schemata that are mainly associated with activity in areas of the SN and subcortical regions such as the amygdala or the striatum are suggested to bias attention, processing, and memory (for review Disner et al., 2011). For example, increased synchronous activity between the striatum and the anterior cingulate cortex is thought to increase both the activity within the mPFC and to decrease the activity within the DLPFC, being associated with biased thinking and memory, leading to depressive symptoms such as rumination. Since these regions are key regions of SN, DMN, and CEN, a link between neurocognitive models of negative bias and aberrant large-scale intrinsic network interactions arises. We suggest an essential relationship between the negative bias model of Beck and the frame of interacting SN, DMN, and CEN (Menon, 2011; Hamilton et al., 2013). In more detail, in the frame of interacting networks [i.e., the triple network model of Menon (2011)] the DMN is involved in self-referential processes (Buckner et al., 2008), while the CEN is involved in goal-directed processes (Fox and Raichle, 2007). The right AI within the SN has been demonstrated to mediate the switching between DMN-based self-referential and CEN-based goal-directed processes (Sridharan et al., 2008). Therefore, anterior insular dysfunction within the SN might contribute to an abnormal switching between DMN and CEN, thus leading to an abnormal behavioral response to both internal and external stimuli and events (Menon and Uddin, 2010). While increased activity in the DMN has been demonstrated to be related with depressive ruminations (Hamilton et al., 2013), abnormal engagement and disengagement of DMN and CEN might underlie difficulties to disengage the processing of negative information, thus negatively biasing attention and cognitive processing. This bias, in turn, might contribute to the worsening of symptoms in patients with MDD. This argument suggests a link between modern large-scale network theories and traditional cognitive theories in MDD, possibly providing a valuable contribution to the better understanding of the neurobiology of MDD. However, several limitations regarding this model have to be considered. We mention only two of them: (i) how are findings beyond the DMN, SN and CEN linked to this model (Williamson and Allman, 2012)? For instance, we found recently that the functional connectivity between both amygdala and hippocampus into the AI and dorsomedial PFC (overlapping with CEN and SN) is consistently disrupted in major depression, potentially constituting a pathway to modulate aberrantly large-scale networks (Tahmasian et al., 2013). (ii) How are DMN, SN, and CEN linked to potential neurochemical anomalies, such as aberrant availability of striatal dopamine in MDD (Nestler and Carlezon, 2006)? For example disrupted reward learning in major depression is associated with the altered reward prediction error activity in the putamen, which again depends on dopaminergic input (Robinson et al., 2012); putamen activity and reward learning are critically linked with the SN (Kapur, 2003), however, it is unknown how these processes relate with the regulation of large-scale networks. Further studies are necessary to test these questions.

### Limitations

We acknowledge several limitations of the current study. (1) Independent Component Analysis. Although often performed, ICA is still associated with methodological constraints, such as arbitrary selection of the model order and subjective bias in identification of the components of interest (Cole et al., 2010). First, our selection of model order was empirical. While model order of around 75 components appears to be optimal for network stability (Abou-Elseoud et al., 2010), computational or objective criteria are still missing. Second, visual selection of networks-of-interest from ICA-derived components has some pitfalls due to subjective bias. To circumvent this problem, we run automated spatial regression analysis of all ICs on network templates from a previous study using the exactly same analysis approach based on a large sample of 603 healthy subjects (Allen et al., 2011). Third, although our results of inter-network connectivity match previous literature, the nature of the interaction within and between different intrinsic connectivity networks or their subsystems is not yet fully understood. For example, Smith et al. (2012), using high-temporal resolution rs-fMRI, discovered remarkable temporal dynamic within intrinsic networks, which is incompletely addressed by our measure of inter-iFC. (2) Causality and inter-iFC between SN and DMN: While we found significantly aberrant inter-iFC between SN and DMN, we did not provide direct evidence for the direction—or stronger causality—of this aberrant interaction. Even when previous studies do suggest a controlling effect from the AI of the SN onto the DMN, our data do not provide direct evidence for this direction of effect. To overcome this missing evidence, the use of Granger causality (GC) analysis (which is a form of time-lagged correlation analysis for BOLD time series) might be a candidate; however, there is still ongoing debate whether BOLD-based GC is a valid method to detect causality among neural processes underlying BOLD signals [for detailed discussion see Manoliu et al. (2013b)]. However, several methods have recently been proposed to overcome the potential restrictions of previous variants of GC analysis (Ryali et al., 2011; Tang et al., 2012). For example Ryali and colleagues proposed a multivariate dynamical systems model (MDS) approach, in which they use the frame of probabilistic graphs to estimate dynamic interactions among regions (Ryali et al., 2011). Definitely, future studies, which apply such advanced methods, are necessary to specify directions of aberrant inter-iFC of the salience network in major depression. (3) Structural anomalies. Since structural anomalies have been shown to have an influence on FC (e.g., Lu et al., 2011), we used the total GM volume extracted from a mask covering the DMN, SN, and CEN as a covariate of no-interest in all statistical analyses of FC. Furthermore, we correlated the GM values derived from the mask covering all 7 ICNs of interest with both inter-iFC between all networks and intra-iFC in the left and right AI within the SN, finding no significant results. However, it is important to note that correcting for linear covariates or investigating potential linear correlations does not exclude the presence of non-linear effects possibly associated with structural changes. Moreover, the effect of structural anomalies on FC is still subject of current research and not yet fully understood. Further studies are necessary to investigate the relationship between structural anomalies and functional connectivity in both healthy participants and patients with MDD. (4) Medication. Antidepressant medication has been demonstrated to have an impact on intrinsic functional connectivity (Delaveau et al., 2011). However, 24 out of 25 patients were medicated at the timepoint of scanning. Although our results are widely consistent with the current literature, the presented data should be interpreted cautiously until replicated in an unmedicated patient sample. (5) Co-morbidities. We would like to point out that although MDD was the primary diagnosis for our patient sample, 14 out of 25 patients with MDD were diagnosed with psychiatric co-morbidities, including generalized anxiety disorder, somatization disorder and avoidant or dependent personality disorder. However, since we aimed to investigate the relationship between intra-iFC, inter-iFC and severity of symptoms in MDD, which is a heterogeneous mental disorder including a high variance in duration of the disorder, number of episodes, family history of MDD and psychiatric co-morbidities, we followed the selection criteria reported in Hennings et al. (2009) to obtain a clinically representative sample, thus including the patients with aforesaid co-morbidities. (6) Although resting-state fMRI is applied frequently throughout the literature to explore possible differences in the brain's functional architecture of patients with mental disorders compared to healthy controls, it is still unclear if the reported alterations might have been at least partially induced by psychological-behavioral differences during the scan. For example, patients might display a higher level of arousal and/or anxiety that, in turn, might have an influence on the ongoing cognitive processes during the scan. In the current study, we investigated whether the patients had experiences of odd feelings during the scan. However, we missed to explicitly measure these possible confounds on a psychometrical or physiological level (e.g., via electrodermatoactivity). Therefore, the possibility that different levels of arousal and/or anxiety might have had an influence on the presented results cannot be excluded.

## Conclusion

The current study provides evidence that aberrant connectivity in the right anterior insula (rAI) within the salience network is associated with the severity of symptoms and aberrant interactions between DMN and CEN in MDD. Data suggest a link between anterior insular dysfunction, altered inter-network connectivity, and severity of symptoms in patients with MDD.

### Conflict of interest statement

The authors declare that the research was conducted in the absence of any commercial or financial relationships that could be construed as a potential conflict of interest.
